# Smooth Approximation *l*_0_-Norm Constrained Affine Projection Algorithm and Its Applications in Sparse Channel Estimation

**DOI:** 10.1155/2014/937252

**Published:** 2014-03-26

**Authors:** Yingsong Li, Masanori Hamamura

**Affiliations:** Graduate School of Engineering, Kochi University of Technology, Kami-shi 782-8502, Japan

## Abstract

We propose a smooth approximation *l*_0_-norm constrained affine projection algorithm (SL0-APA) to improve the convergence speed and the steady-state error of affine projection algorithm (APA) for sparse channel estimation. The proposed algorithm ensures improved performance in terms of the convergence speed and the steady-state error via the combination of a smooth approximation *l*_0_-norm (SL0) penalty on the coefficients into the standard APA cost function, which gives rise to a zero attractor that promotes the sparsity of the channel taps in the channel estimation and hence accelerates the convergence speed and reduces the steady-state error when the channel is sparse. The simulation results demonstrate that our proposed SL0-APA is superior to the standard APA and its sparsity-aware algorithms in terms of both the convergence speed and the steady-state behavior in a designated sparse channel. Furthermore, SL0-APA is shown to have smaller steady-state error than the previously proposed sparsity-aware algorithms when the number of nonzero taps in the sparse channel increases.

## 1. Introduction

With the development of wireless communication, there have been increasing demands for higher transmission rates in modern communication systems. This has led to the development of new standards for various wireless devices, such as smartphones, laptops, and iPads [1–5]. Given these requirements, broadband signal transmission is an essential technique for next-generation wireless communication systems [6]. In broadband wireless communications, a “hilly terrain” (HT) delay profile consists of a sparsely distributed multipath channel in which most of taps are zero or close to zero, while only a few taps are dominant [4]. In this paper, we consider the communication problems which involve the estimation and equalization of channels with a large delay spread but with a small nonzero support, which is also known as sparse channel estimation.

Recently, a rising method for sparse channel estimation has been proposed and extensively investigated by the use of compressed sensing (CS) to improve the performance of such sparse wireless communication channels [7–9]. We found that these CS channel estimation algorithms were sensitive to the channel interferences. Another effective class of methods that have been widely studied in channel estimation is adaptive filtering algorithms [10–13], such as least mean square (LMS), recursive least squares (RLS), and Kalman filter algorithms. However, these standard adaptive filtering algorithms cannot utilize the sparse property of the wireless communication channel and hence they perform poorly in dealing with the sparse signals. To utilize the sparse characteristic of such channels, some improved adaptive filtering algorithms by the use of partial updating techniques have been proposed and investigated in wireless communications [14–16]. However, this partial updating degraded the estimation performance in contrast to the standard LMS and RLS algorithms.

Motivated by the widely developed CS techniques [17, 18], some efforts have been put into combining the CS technique into the adaptive filtering algorithms in order to improve the performance of standard adaptive filtering performance for sparse signal recovery. For example, a Kalman filter compressed sensing (KF-CS) algorithm has been proposed and applied in magnetic resonance imaging (MRI) by the combination of CS and standard Kalman filter [19]. In this algorithm, Kalman filter estimates the support set which has significant effect on the estimator errors. Furthermore, another algorithm denoted as least square compressed sensing (LS-CS) has been developed and well investigated by using the CS and RLS techniques [20, 21]. Unfortunately, these algorithms are highly complex because of the computational complexity of Kalman filter and RLS algorithms. LMS algorithm has attracted much more attention in recent years due to its low computational complexity and reliable recovery capability. Inspired by the CS theory [17, 18] and the KF-CS and LS-CS algorithms, several sparsity-aware LMS algorithms have been proposed with additional norm constrained terms in the cost function of standard LMS algorithms [6, 22–27]. It was found in these studies that these linear constrained sparsity-aware LMS algorithms can achieve faster convergence speed and better steady-state performance compared to the standard LMS algorithm. However, these sparsity-aware LMS algorithms are sensitive to the noise and the sparsity characteristics of the channel, which results in high steady-state misadjustment due to the estimation error that occurs in the adaptation. The affine projection algorithm (APA) is another popular method in adaptive filtering applications [28–31], with its complexity and estimation performance intermediary between the LMS and RLS algorithms. The APA reuses old data resulting in fast convergence, and is also an improved normalized LMS (NLMS) algorithm that converges faster than the standard LMS algorithm. Subsequently, *l*_1_-norm penalized APA has been proposed to render the standard APA suitable for sparse signal estimation applications [32]. However, these *l*_1_-norm penalized APAs impose the condition that the proportion of nonzero taps must be very small as compared to the proportion of dominant taps in the associated parameter vector in channel estimation.

In this paper, we propose a smooth approximation *l*_0_-norm constrained affine projection (SL0-APA) algorithm for sparse channel estimation. The proposed SL0-APA is similar to the algorithms proposed in [32], which are known as zero-attracting affine projection algorithm (ZA-APA) and reweighted zero-attracting affine projection algorithm (RZA-APA). It differs by the regularization term which is a smooth approximation *l*_0_-norm obtained from a continuous function that is an accurate approximation of *l*_0_-norm. By exploiting the information of the sparsity channel and using the concepts of the smooth approximation of *l*_0_-norm, we can improve the performance of the previous sparsity-aware APAs with respect to both the convergence speed and the steady-state performance. We also provide a convergence analysis and the mean-square-error analysis of our proposed SL0-APA. Furthermore, we experimentally investigate the effect of adding a smooth approximation *l*_0_-norm penalty term to the cost function on learning the convergence behavior and the steady-state error performance of the SL0-APA. Accordingly, we experimentally illustrate that the SL0-APA is superior to ZA-APA and RZA-APA in terms of steady-state error and the convergence speed. Besides, the theoretical analysis is also presented and compared to the computer simulation results. Finally, the computational complexity of the proposed SL0-APA is mathematically given and is experimentally evaluated.

The remainder of the paper is structured as follows.  Section 2 briefly reviews the standard APA, ZA-APA, and RZA-APA based on a sparse multipath communication system. In Section 3, we first propose a SL0-APA by the use of a smooth approximation *l*_0_-norm penalty on the cost function of the standard APA. Next, we provide a theoretical expression of the convergence analysis and the mean-square-error (MSE) analysis of our proposed SL0-APA based on the energy-conservation approach. In Section 4, the proposed SL0-APA is experimentally investigated over a sparse channel to demonstrate the estimation performance of the SL0-APA, including the convergence speed, steady-state error, and the computational complexity. Finally, Section 5 is the conclusion.

## 2. Conventional Channel Estimation Algorithms

**Figure 1:**
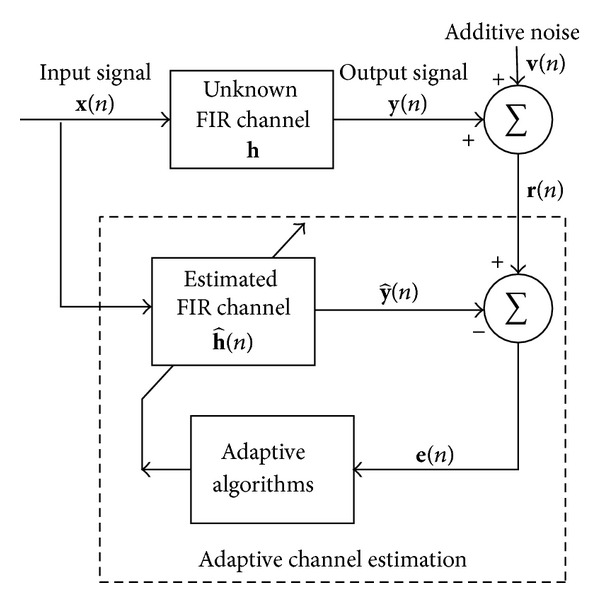
A typical sparse multipath communication system.

In this section, we consider a sparse multipath communication system shown in Figure 1 to discuss traditional channel estimation algorithms. The input signal **x**(*n*) = [*x*(*n*), *x*(*n* − 1),…, *x*(*n* − *N* + 1)]^*T*^ containing the *N* most recent samples is transmitted over a finite impulse response (FIR) channel with channel impulse response (CIR) **h** = [*h*_0_, *h*_1_,…, *h*_*N*−1_]^*T*^, where (·)^*T*^ denotes the transposition. The input signal **x**(*n*) is also used as an input for an adaptive filter $\hat{h}(n)$ with *N* coefficients to produce an estimation output $\hat{y}(n)$, and the received signal **r**(*n*) = **y**(*n*) + **v**(*n*) is obtained at the receiver.

### 2.1. Affine Projection Algorithm (APA)

The channel estimation technique called the standard APA estimates the unknown sparse channel **h** using the input signal **x**(*n*) and the output signal **y**(*n*). In the standard APA, let us assume that we keep the last *Q* input signal **x**(*n*) to form the matrix **U**(*n*) as follows [28]:

(1) U(n)=[xT(n)xT(n−1)⋮xT(n−Q+1)]=[x(n)x(n−1)⋯x(n−N+1)x(n−1)x(n−2)⋯x(n−N)⋮⋮⋱⋮x(n−Q+1)x(n−Q)⋯x(n−N−Q+2)],

where *Q* denotes the projection order of the APA. Furthermore, we also define some vectors representing reusing results at a given instant *n*, such as the output **y**(*n*) of the channel, the output $\hat{y}(n)$ of the filter, the received signal **r**(*n*), and the additive white Gaussian noise vector **v**(*n*) and these vectors are expressed as

(2) $$\begin{array}{c} y\left(n\right)=U\left(n\right)h=\left[\begin{array}{c} y\left(n\right)\\ y\left(n-1\right)\\ \vdots \\ y\left(n-Q+1\right) \end{array}\right], \end{array}$$

(3) $$\begin{array}{c} \hat{y}\left(n\right)=U\left(n\right)\hat{h}\left(n\right)=\left[\begin{array}{c} \hat{y}\left(n\right)\\ \hat{y}\left(n-1\right)\\ \vdots \\ \hat{y}\left(n-Q+1\right) \end{array}\right], \end{array}$$

(4) $$\begin{array}{c} v\left(n\right)=\left[\begin{array}{c} v\left(n\right)\\ v\left(n-1\right)\\ \vdots \\ v\left(n-Q+1\right) \end{array}\right], \end{array}$$

(5) $$\begin{array}{c} r\left(n\right)=\left[\begin{array}{c} r\left(n\right)\\ r\left(n-1\right)\\ \vdots \\ r\left(n-Q+1\right) \end{array}\right]. \end{array}$$

From (1)–(5), the instantaneous error **e**(*n*) can be written as

(6) e(n)=[e(n)e(n−1)⋮e(n−Q+1)]=[r(n)−y^(n)r(n−1)−y^(n−1)⋮r(n−Q+1)−y^(n−Q+1)]=r(n)−y^(n).

As for the channel estimation, the purpose of the APA is to minimize

(7) ||h^(n+1)−h^(n)||2subject  to:r(n)−U(n)h^(n+1)=0.

The APA maintains the next coefficient $\hat{h}(n+1)$ as close as possible to the current coefficient $\hat{h}(n)$ and minimizes the a posteriori error to zero at the same time. Here, the Lagrange multiplier is used to find out the solution that minimizes the cost function *J*_APA_(*n*) of the APA:

(8) JAPA(n)=||h^(n+1)−h^(n)||2+[r(n)−U(n)h^(n+1)]TλAPA,

where ***λ***_APA_ is a *Q* × 1 vector of Lagrange multiplier and $\lambda_{\text{APA}}={\left[\begin{array}{cccc} \lambda_{0} & \lambda_{1} & \cdots & \lambda_{Q-1} \end{array}\right]}^{T}$. Equation (8) can be rewritten as

(9) JAPA(n)=[h^(n+1)−h^(n)]T[h^(n+1)−h^(n)]+[rT(n)−h^T(n+1)UT(n)]λAPA.

Then, the gradient of *J*_APA_(*n*) with respect to $\hat{h}(n+1)$ is given by

(10) $$\begin{array}{c} \frac{\partial J_{\text{APA}}\left(n\right)}{\partial \hat{h}\left(n+1\right)}=2\hat{h}\left(n+1\right)-2\hat{h}\left(n\right)-U^{T}\left(n\right)\lambda_{\text{APA}}. \end{array}$$

After setting the gradient of *J*_APA_(*n*) with respect to $\hat{h}(n+1)$ equal to zero, we get

(11) $$\begin{array}{c} \hat{h}\left(n+1\right)=\hat{h}\left(n\right)+\frac{1}{2}U^{T}\left(n\right)\lambda_{\text{APA}}. \end{array}$$

Multiplying **U**(*n*) on both sides of (11), we have

(12) $$\begin{array}{c} U\left(n\right)\hat{h}\left(n+1\right)=U\left(n\right)\hat{h}\left(n\right)+\frac{1}{2}U\left(n\right)U^{T}\left(n\right)\lambda_{\text{APA}}. \end{array}$$

By taking the constraint condition of (7) into consideration, we have

(13) $$\begin{array}{c} r\left(n\right)=U\left(n\right)\hat{h}\left(n\right)+\frac{1}{2}U\left(n\right)U^{T}\left(n\right)\lambda_{\text{APA}}. \end{array}$$

Taking (3), (6), and (12) into account, we can get

(14) $$\begin{array}{c} e\left(n\right)=\frac{1}{2}U\left(n\right)U^{T}\left(n\right)\lambda_{\text{APA}}. \end{array}$$

Then

(15) $$\begin{array}{c} \lambda_{\text{APA}}=2{\left[U\left(n\right)U^{T}\left(n\right)\right]}^{-1}e\left(n\right). \end{array}$$

The update equation is now given by (11) with ***λ***_APA_ being the solution of (14) and is expressed as

(16) h^(n+1)=h^(n)+UT(n)[U(n)UT(n)]−1e(n)=h^(n)+U+(n)e(n),

where **U** ^+^(*n*) = **U** ^*T*^(*n*)[**U**(*n*)**U** ^*T*^(*n*)]^−1^. The above update equation corresponds to the conventional APA with unity convergence factor [28]. In the practical engineering applications, a convergence factor *μ*_APA_, also known as step-size, is adopted to tradeoff the mean square misadjustment and convergence speed, and thus, the update equation (16) can be rewritten as

(17) h^(n+1)=h^(n)+μAPAUT(n)[U(n)UT(n)]−1e(n)=h^(n)+μAPAU+(n)e(n).

In general, the step-size *μ*_APA_ should be chosen in the range 0 < *μ*_APA_ < 2 to control the convergence speed and the steady-state behavior of the APA. It is worth noting that the APA becomes familiar normalized least mean square (NLMS) when the *Q* = 1.

### 2.2. Zero-Attracting Affine Projection Algorithm (ZA-APA)

To improve the performance of the standard APA and to utilize the sparsity property of the sparse multipath communication channel, an *l*_1_-penalty term is cooperated into the cost function of (8), which is known as zero-attracting affine projection algorithm (ZA-APA) [32]. In the ZA-APA, the cost function is defined by combining the cost function *J*_APA_(*n*) of standard APA with *l*_1_-penalty of the channel estimator and is given by

(18) JZA(n)=||h^(n+1)−h^(n)||2+[r(n)−U(n)h^(n+1)]TλZA+γZA||h^(n+1)||1,

where ***λ***_ZA_ is the vector of Lagrange multiplier with *Q* × 1. *γ*_ZA_ > 0 is a regularization parameter to balance the estimation error and the sparse *l*_1_-penalty of $\hat{h}(n+1)$. In order to minimize the cost function *J*_ZA_(*n*), we use the Lagrange multiplier to calculate its gradient, which is expressed as

(19) ∂JZA(n)∂h^(n+1)=2h^(n+1)−2h^(n)−UT(n)λZA+γZAsgn⁡[h^(n+1)],

where sgn⁡[·] is a component-wise sign function defined as

(20) $$\begin{array}{c} \mathrm{sgn}\left[x\right]=\{\begin{array}{cc} \frac{x}{\left|x\right|}, & x\neq 0\\ 0, & x=0. \end{array} \end{array}$$

As is known to us, the minimum is obtained by letting $\partial J_{\text{ZA}}(n)/\partial \hat{h}(n+1)=0$. Thus, we can get

(21) $$\begin{array}{c} \hat{h}\left(n+1\right)=\hat{h}\left(n\right)+\frac{1}{2}U^{T}\left(n\right)\lambda_{\text{ZA}}-\frac{1}{2}\gamma_{\text{ZA}}\mathrm{sgn}\left[\hat{h}\left(n+1\right)\right]. \end{array}$$

Multiplying both sides by **U**(*n*), we can obtain

(22) U(n)h^(n+1)=U(n)h^(n)+12U(n)UT(n)λZA−12γZAU(n)sgn⁡[h^(n+1)].

Considering the constraint condition of (7), we can get the following expression:

(23) r(n)=U(n)h^(n)+12U(n)UT(n)λZA−12γZAU(n)sgn⁡[h^(n+1)].

From the above discussion, we know that $e(n)=r(n)-U(n)\hat{h}(n)$. Thus, the Lagrange multipliers vector ***λ***_ZA_ is obtained:

(24) $$\begin{array}{c} \lambda_{\text{ZA}}={\left[U\left(n\right)U^{T}\left(n\right)\right]}^{-1}\left\{2e\left(n\right)+\gamma_{\text{ZA}}U\left(n\right)\mathrm{sgn}\left[\hat{h}\left(n+1\right)\right]\right\}. \end{array}$$

Substituting (24) into (21) and assuming that $\mathrm{sgn}[\hat{h}(n+1)]\approx \mathrm{sgn}[\hat{h}(n)]$, we can obtain the update function of the ZA-APA:

(25) h^(n+1)=h^(n)+U+(n)e(n)+12γZAU+(n)U(n)sgn⁡[h^(n)]−12γZAsgn⁡[h^(n)].

To balance the convergence speed and steady-state error, a step-size *μ*_ZA_ is introduced and integrated into (25). Then, (25) can be rewritten as

(26) h^(n+1)=h^(n)+μZAU+(n)e(n)+12γZAU+(n)U(n)sgn⁡[h^(n)]−12γZAsgn⁡[h^(n)].

Comparing the update equation (26) of the ZA-APA with the update (17) of the standard APA, we find that there are two additional terms in (26) which attract the tap coefficients to zero when the tap magnitudes of the sparse channel are close to zero. These two additional terms are zero attractors whose attracting strengths are controlled by *γ*_ZA_. Intuitively, the zero attractor can speed the convergence of ZA-APA when the majority taps of the channel of **h** are zero or close to zero, such as sparse channel.

### 2.3. Reweighted Zero-Attracting Affine Projection Algorithm (RZA-APA)

Unfortunately, the ZA-APA cannot distinguish the zero taps and the nonzero taps of the sparse channel, and it exerts the same penalty on all the channel taps, which forces all the taps to zero uniformly [22, 32]. Therefore, the performance of the ZA-APA is degraded when the channel is a less sparse one. In order to improve the performance of the ZA-APA and to solve this problem, a heuristic approach first reported in [33] and employed in [22, 32] to reinforce that the zero attractor was proposed and was denoted as reweighted zero-attracting affine projection algorithm (RZA-APA). In the RZA-APA, $\sum_{i=1}^{N}\log(1+\varepsilon_{\text{RZA}}\left|{\hat{h}}_{i}(n)\right|)$ is adopted instead of ${||\hat{h}(n)||}_{1}$. Thus, the cost function of the RZA-APA can be written as

(27) JRZA(n)=||h^(n+1)−h^(n)||2+[r(n)−U(n)h^(n+1)]TλRZA+γRZA∑i=1Nlog⁡(1+εRZA|h^i(n+1)|),

where *γ*_RZA_ > 0 is a regularization parameter, *ε*_RZA_ > 0 is a positive threshold, and ***λ***_RZA_ is the vector of the Lagrange multiplier with size of *Q* × 1. The Lagrange multiplier is used for calculating the minimization of *J*_RZA_(*n*) and the gradient of *J*_RZA_(*n*) can be expressed as

(28) ∂JRZA(n)∂h^(n+1)=2h^(n+1)−2h^(n)−UT(n)λRZA+γRZAsgn⁡[h^(n+1)]1+εRZA|h^(n+1)|.

Let $\partial J_{\text{RZA}}(n)/\partial \hat{h}(n+1)=0$ and assume $\mathrm{sgn}[\hat{h}(n+1)]/(1+\varepsilon_{\text{RZA}}\left|\hat{h}(n+1)\right|)\approx \mathrm{sgn}[\hat{h}(n)]/(1+\varepsilon_{\text{RZA}}\left|\hat{h}(n)\right|)$, and then we can get

(29) $$\begin{array}{c} \hat{h}\left(n+1\right)=\hat{h}\left(n\right)+\frac{1}{2}U^{T}\left(n\right)\lambda_{\text{RZA}}-\frac{1}{2}\gamma_{\text{RZA}}\frac{\mathrm{sgn}\left[\hat{h}\left(n\right)\right]}{1+\varepsilon_{\text{RZA}}\left|\hat{h}\left(n\right)\right|}. \end{array}$$

By multiplying **U**(*n*) on both sides of (29), the following equation can be obtained:

(30) U(n)h^(n+1)=U(n)h^(n)+12U(n)UT(n)λRZA−12γRZAU(n)sgn⁡[h^(n)]1+εRZA|h^(n)|.

Taking (7) and (30) into consideration, we can get

(31) r(n)=U(n)h^(n)+12U(n)UT(n)λRZA−12γRZAU(n)sgn⁡[h^(n)]1+εRZA|h^(n)|.

Thus, the Lagrange multiplier vector ***λ***_RZA_ is obtained:

(32) λRZA=[U(n)UT(n)]−1×{2e(n)+γRZAU(n)sgn⁡[h^(n)]1+εRZA|h^(n)|},

where $e(n)=r(n)-U(n)\hat{h}(n)$. Substituting (32) into (29), we can get the update equation of the RZA-APA:

(33) h^(n+1)=h^(n)+U+(n)e(n)+12γRZAU+(n)U(n)sgn⁡[h^(n)]1+εRZA|h^(n)|−12γRZAsgn⁡[h^(n)]1+εRZA|h^(n)|.

Similarly, a step-size *μ*_RZA_ is introduced and cooperated into (33) to balance the convergence speed and the steady-state error of the RZA-APA. Then, (33) can be rewritten as

(34) h^(n+1)=h^(n)+μRZAU+(n)e(n)+12γRZAU+(n)U(n)sgn⁡[h^(n)]1+εRZA|h^(n)|−12γRZAsgn⁡[h^(n)]1+εRZA|h^(n)|.

From the analysis and the a priori knowledge of the sparse channel, we know that the RZA-APA is more sensitive to taps with small magnitudes. Note that the reweighted zero attractor mainly affects taps whose magnitudes are comparable to 1/*ε*_RZA_ while it has less shrinkage exerted on $\left|\hat{h}(n)\right|\gg 1/\varepsilon_{\text{RZA}}$. Thus, the RZA-APA can improve steady-state performance compared to the ZA-APA.

## 3. Proposed Smooth Approximation *l*_0_-Norm Constrained Affine Projection Algorithm (SL0-APA)

On the basis of the discussion of the ZA-APA and RZA-APA, we find that the RZA-APA can improve the performance of ZA-APA for sparse channel estimation because $\sum_{i=1}^{N}\log(1+\varepsilon_{\text{RZA}}\left|{\hat{h}}_{i}(n+1)\right|)$ is more similar to *l*_0_-norm [22, 32, 33]. On the other hand, solving *l*_0_-norm ${||\hat{h}(n+1)||}_{0}$ is a NP-hard problem [18]. Fortunately, smooth approximation *l*_0_-norm (SL0) with low complexity has been proposed as an accurate approximation of ${||\hat{h}(n+1)||}_{0}$ to reconstruct sparse signals in CS theory [34, 35]. Inspired by the SL0 algorithm and in order to exploit the sparse characteristic of the multipath channel in a more accurate way, a smooth approximation *l*_0_-norm constrained affine projection algorithm (SL0-APA) is proposed by exerting the SL0 on the cost function of standard APA to further improve the performance of the RZA-APA.

### 3.1. Proposed SL0-APA

Similar to the ZA-APA and RZA-APA discussed above, the cost function of the SL0-APA is written as

(35) JSL0(n)=||h^(n+1)−h^(n)||2+[r(n)−U(n)h^(n+1)]TλSL0+γSL0||h^(n+1)||0,

where ***λ***_SL0_ is the vector of the Lagrange multiplier with size of *Q* × 1 and *γ*_SL0_ > 0 is a regularization parameter to tradeoff the estimation error and the sparse *l*_0_-penalty of $\hat{h}(n+1)$. Here, the smooth approximation of *l*_0_-norm ${||\hat{h}(n+1)||}_{0}$ is a continuous function defined as follows:

(36) $$\begin{array}{c} {||\hat{h}\left(n+1\right)||}_{0}=\overset{N-1}{\underset{i=1}{\sum }}\frac{\left|{\hat{h}}_{i}\left(n+1\right)\right|}{\left|{\hat{h}}_{i}\left(n+1\right)\right|+\delta }=\frac{\left|\hat{h}\left(n+1\right)\right|}{\left|\hat{h}\left(n+1\right)\right|+\delta }, \end{array}$$

where *δ* is a small positive constant which is used for avoiding division by zero, and the gradient of this continuous functions for SL0 is obtained:

(37) $$\begin{array}{c} \frac{\partial {||\hat{h}\left(n+1\right)||}_{0}}{\partial \hat{h}\left(n+1\right)}=\frac{\delta \mathrm{sgn}\left(\hat{h}\left(n+1\right)\right)}{{\left(\left|\hat{h}\left(n+1\right)\right|+\delta \right)}^{2}}. \end{array}$$

To obtain the minimum of the *J*_SL0_(*n*), we use Lagrange multiplier to calculate the gradient of *J*_SL0_(*n*). Then the gradient of the cost function of the SL0-APA is written as

(38) ∂JSL0(n)∂h^(n+1)=2h^(n+1)−2h^(n)−UT(n)λSL0+γSL0δsgn⁡(h^(n+1))(|h^(n+1)|+δ)2.

Let the left-hand side of (38) be equal to zero. We can get the following equation:

(39) $$\begin{array}{c} \hat{h}\left(n+1\right)=\hat{h}\left(n\right)+\frac{1}{2}U^{T}\left(n\right)\lambda_{\text{SL}0}-\frac{1}{2}\gamma_{\text{SL}0}\frac{\delta \mathrm{sgn}\left(\hat{h}\left(n+1\right)\right)}{{\left(\left|\hat{h}\left(n+1\right)\right|+\delta \right)}^{2}}. \end{array}$$

Multiplying **U**(*n*) on both sides of (39), we can get

(40) U(n)h^(n+1)=U(n)h^(n)+12U(n)UT(n)λSL0−12γSL0U(n)δsgn⁡(h^(n+1))(|h^(n+1)|+δ)2.

By taking (7) into consideration, (40) can be rewritten as

(41) r(n)=U(n)h^(n)+12U(n)UT(n)λSL0−12γSL0U(n)δsgn⁡(h^(n+1))(|h^(n+1)|+δ)2.

From the discussion of the ZA-APA and RZA-APA, we can get the Lagrange multiplier vector ***λ***_SL0_ from (41) by taking $e(n)=r(n)-U(n)\hat{h}(n)$ into account:

(42) λSL0=[U(n)UT(n)]−1×{2e(n)+γSL0U(n)δsgn⁡(h^(n+1))(|h^(n+1)|+δ)2}.

Substituting (42) into (39) and assuming that $\delta \mathrm{sgn}{\left(\hat{h}(n+1))/(\left|\hat{h}(n+1)\right|+\delta \right)}^{2}\approx \delta \mathrm{sgn}(\hat{h}(n))/{\left(\left|\hat{h}(n)\right|+\delta \right)}^{2}$, the update function of the SL0-APA can be achieved:

(43) h^(n+1)=h^(n)+U+(n)e(n)+12γSL0U+(n)U(n)δsgn⁡(h^(n))(|h^(n)|+δ)2−12γSL0δsgn⁡(h^(n))(|h^(n)|+δ)2=h^(n)+U+(n)e(n)+12γSL0U+(n)U(n)T(n)−12γSL0T(n),

where $T(n)=\delta \mathrm{sgn}(\hat{h}(n))/{\left(\left|\hat{h}(n)\right|+\delta \right)}^{2}$. Similar to the ZA-APA and RZA-APA, a step-size *μ*_SL0_ is introduced into (43) to create a balance between the convergence speed and steady-state error of the SL0-APA:

(44) h^(n+1)=h^(n)+μSL0U+(n)e(n)+12γSL0U+(n)U(n)T(n)−12γSL0T(n).

It is important to mention that our proposed SL0-APA is superior to APA, ZA-APA, and RZA-APA for sparse channel estimation because we utilize a smooth approximation of ${||\hat{h}(n+1)||}_{0}$, which is proved to be an approximate and near-accurate approximation of *l*_0_-norm in comparison with the sum-log function $\sum_{i=1}^{N}\log(1+\varepsilon_{\text{RZA}}\left|{\hat{h}}_{i}(n+1)\right|)$ in the RZA-APA. Moreover, it is easy to calculate the gradient, as we can easily find a continuous gradient for this smoothed *l*_0_-norm function.

### 3.2. Analysis of the Proposed SL0-APA

In this section, we analyze the mean-square-error (MSE) behavior of the SL0-APA. Here, energy-conservation approach [36–38] is employed to obtain the theoretical expressions for the MSE of the SL0-APA. Let us consider the received signal **r**(*n*) that is derived from the following linear model:

(45) $$\begin{array}{c} r\left(n\right)=U\left(n\right)h+v\left(n\right), \end{array}$$

where **h** is the sparse channel vector of the multipath communication system that we wish to estimate and **v**(*n*) is the additive Gaussian noise at instant *n*. Our objective is to evaluate the steady-state MSE performance of the proposed SL0-APA. The steady-state MSE is defined as

(46) $$\begin{array}{c} \text{MSE}≜\underset{n\to \infty }{\lim}\text{E}\left[{\left|e\left(n\right)\right|}^{2}\right], \end{array}$$

where E[·] denotes the expectation and

(47) $$\begin{array}{c} e\left(n\right)=r\left(n\right)-U\left(n\right)\hat{h}\left(n\right) \end{array}$$

is the estimated error at time *n*. Taking (45) and (47) into account, we obtain

(48) e(n)=U(n)h+v(n)−U(n)h^(n)=U(n)[h−h^(n)]+v(n).

Subtracting **h** from both sides of the SL0-APA update function (44), we get the misalignment vector:

(49) Δ(n+1)=h−h^(n+1)=h−{h^(n)+μSL0U+(n)e(n)+12γSL0U+(n)U(n)T(n)−12γSL0T(n)}.

Substituting (48) into (49), we can get

(50) Δ(n+1)=h−h^(n)−μSL0U+(n)×{U(n)[h−h^(n)]+v(n)}−12γSL0U+(n)U(n)T(n)+12γSL0T(n)=[IN−μSL0U+(n)U(n)]Δ(n)−μSL0U+(n)v(n)−12γSL0U+(n)U(n)T(n)+12γSL0T(n).

Taking expectations on both sides of (50), we get

(51) E[Δ(n+1)]=E[IN−μSL0U+(n)U(n)]E[Δ(n)]−μSL0E[U+(n)v(n)]−12γSL0E[U+(n)U(n)T(n)]+12γSL0E[T(n)].

We assume that the additive noise **v**(*n*) is statistically independent of the input signal **x**(*n*), and hence we have E[**U** ^+^(*n*)**v**(*n*)] = 0. Therefore, (51) can be simplified as

(52) E[Δ(n+1)]=E[IN−μSL0U+(n)U(n)]E[Δ(n)]−12γSL0E[U+(n)U(n)T(n)]+12γSL0E[T(n)].

From previous studies on sparse LMS algorithms [22, 39], in the steady state, we have

(53) $$\begin{array}{c} \text{E}\left\{\mathrm{sgn}\left[\hat{h}\left(n\right)\right]\right\}\approx \mathrm{sgn}\left(\hat{h}\right). \end{array}$$

Thus, the E[**T**(*n*)] in (52) can be written as

(54) $$\begin{array}{c} \text{E}\left[T\left(n\right)\right]=\text{E}\left\{\frac{\delta \mathrm{sgn}\left(\hat{h}\left(n\right)\right)}{{\left(\left|\hat{h}\left(n\right)\right|+\delta \right)}^{2}}\right\}=\frac{\delta \mathrm{sgn}\left(\hat{h}\left(n\right)\right)}{{\left(\left|\hat{h}\left(n\right)\right|+\delta \right)}^{2}}. \end{array}$$

In addition, when the channel length is far larger than 1, *N* ≫ 1, the E[**U** ^+^(*n*)**U**(*n*)] can be written as [37, 40, 41]

(55) E[U+(n)U(n)]=E{UT(n)[U(n)UT(n)]−1U(n)}≈E{UT(n){E[U(n)UT(n)]}−1U(n)}.

Since E[**x** ^*T*^(*n*)**x**(*n* − 1)] = 0 for sparse channel estimation, the inner expectation reduces to

(56) E[U(n)UT(n)]=E{[xT(n)xT(n−1)⋮xT(n−Q−1)][x(n)x(n−1)⋯x(n−Q−1)]}=E{[||x(n)||2xT(n)x(n−1)⋯xT(n)x(n−Q−1)xT(n−1)x(n)||x(n−1)||2⋯⋮⋮⋮⋱⋮xT(n−Q−1)x(n)xT(n−Q−1)x(n−1)⋯||x(n−Q−1)||2]}=E{[||x(n)||20⋯00||x(n−1)||2⋯0⋮⋮⋱⋮00⋯||x(n−Q−1)||2]}.

Here, we define

(57) $$\begin{array}{c} R=\delta_{x}^{2}I_{N}, \end{array}$$

where *δ*_*x*_^2^ is the power of the input signal. Thus,

(58) $$\begin{array}{c} \text{E}\left[U\left(n\right)U^{T}\left(n\right)\right]\approx \mathrm{Tr}\left(R\right)I_{Q}=N\delta_{x}^{2}I_{Q}, \end{array}$$

where *Tr*⁡(·) is the trace of matrix and **I**_*Q*_ is the *Q* × *Q* identity matrix. Moreover, we can obtain

(59) $$\begin{array}{c} \text{E}\left\{{\left[U\left(n\right)U^{T}\left(n\right)\right]}^{-1}\right\}\approx {\left\{\text{E}\left[U\left(n\right)U^{T}\left(n\right)\right]\right\}}^{-1}=\frac{1}{N\delta_{x}^{2}}I_{Q}. \end{array}$$

Then we can approximate E{**U** ^*T*^(*n*)[**U**(*n*)**U** ^*T*^(*n*)]^−1^ **U**(*n*)} by

(60) E{UT(n)[U(n)UT(n)]−1U(n)}≈E{UT(n)1Nδx2IQU(n)}≈QRNδx2.

Therefore, (52) can be rewritten as

(61) E[Δ(n+1)]=E[IN−μSL0QRNδx2]E[Δ(n)]−12γSL0QRNδx2E[T(n)]+12γSL0E[T(n)].

It is found that the matrix **T**(*n*) is approximately bounded between −*δ* **I**_*N*_ and *δ* **I**_*N*_. Therefore, we see that such convergence is guaranteed only if (**I**_*N*_ − *μ*_SL0_ *Q* **R**/*Nδ*_*x*_^2^) is less than 1 [28], which is given by

(62) $$\begin{array}{c} 0<\mu_{\text{SL}0}<\frac{N\delta_{x}^{2}}{Q\lambda_{\max}}, \end{array}$$

where *λ*_max⁡_ is the maximum eigenvalue of the autocorrelation matrix **R** of **x**(*n*). We can observe that the stability condition of the SL0-APA is independent of the parameter *γ*_SL0_. We assume that the estimated vector $\hat{h}(n)$ converges when *n* → *∞*. Then, (61) can be rewritten as

(63) E[Δ(∞)]=[IN−μSL0QRNδx2]E[Δ(∞)]−12γSL0QRNδx2δsgn⁡(h)(h+δ)2+12γSL0δsgn⁡(h)(h+δ)2.

From (63), we can obtain

(64) $$\begin{array}{c} \text{E}\left[\Delta \left(\infty \right)\right]=-\frac{\gamma_{\text{SL}0}}{2\mu_{\text{SL}0}}\frac{\delta \mathrm{sgn}\left(h\right)}{{\left(h+\delta \right)}^{2}}+\frac{\gamma_{\text{SL}0}}{2\mu_{\text{SL}0}}\frac{N\delta_{x}^{2}}{QR}\frac{\delta \mathrm{sgn}\left(h\right)}{{\left(h+\delta \right)}^{2}}, \end{array}$$

which can be regarded as

(65) $$\begin{array}{c} \text{E}\left[\hat{h}\left(\infty \right)\right]=h-\frac{\gamma_{\text{SL}0}}{2\mu_{\text{SL}0}}\frac{\delta \mathrm{sgn}\left(h\right)}{{\left(h+\delta \right)}^{2}}+\frac{\gamma_{\text{SL}0}}{2\mu_{\text{SL}0}}\frac{N\delta_{x}^{2}}{QR}\frac{\delta \mathrm{sgn}\left(h\right)}{{\left(h+\delta \right)}^{2}}. \end{array}$$

Note that (65) implies that the optimum solution of the SL0-APA is biased, as was also shown for zero-attracting least mean square (ZA-LMS) algorithms [22]. We then proceed to derive the steady-state MSE for our proposed SL0-APA. Firstly, multiplying both sides of (44) by **U**(*n*) from the left, we can get

(66) U(n)h^(n+1)=U(n)h^(n)+μSL0U(n)U+(n)e(n)+12γSL0U(n)U+(n)U(n)T(n)−12γSL0U(n)T(n).

Furthermore,

(67) $$\begin{array}{c} U\left(n\right)\hat{h}\left(n+1\right)=U\left(n\right)\hat{h}\left(n\right)+\mu_{\text{SL}0}e\left(n\right). \end{array}$$

Additionally, we define the a posteriori error vector **e**_*p*_(*n*) and the a priori error vector **e**_*a*_(*n*) as

(68) ep(n)=U(n)h−U(n)h^(n+1)ea(n)=U(n)h−U(n)h^(n).

Combining (67) and (68), we have

(69) $$\begin{array}{c} e_{p}\left(n\right)=e_{a}\left(n\right)-\mu_{\text{SL}0}e\left(n\right). \end{array}$$

In addition,

(70) e(n)=r(n)−U(n)h^(n)=U(n)h+v(n)−U(n)h^(n)=ea(n)+v(n).

By substituting (70) into (69), we have

(71) $$\begin{array}{c} e_{p}\left(n\right)=\left(I-\mu_{\text{SL}0}\right)e\left(n\right)-v\left(n\right). \end{array}$$

From (69), we can also write the **e**(*n*) as follows:

(72) $$\begin{array}{c} e\left(n\right)=\frac{1}{\mu_{\text{SL}0}}\left[e_{a}\left(n\right)-e_{p}\left(n\right)\right]. \end{array}$$

Substituting (72) to (44), we have

(73) h^(n+1)=h^(n)+U+(n)[ea(n)−ep(n)]+12γSL0U+(n)U(n)T(n)−12γSL0T(n).

On the basis of the discussion mentioned above, we notice that **U**(*n*)**U** ^+^(*n*) = **U**(*n*)**U** ^*T*^(*n*)[**U**(*n*)**U** ^*T*^(*n*)]^−1^ = **I**. By considering the power of both sides of (73), using the steady-state condition $\text{E}\left[{||\hat{h}(n+1)||}^{2}\right]\approx \text{E}\left[{||\hat{h}(n)||}^{2}\right]$ when *n* → *∞*, and assuming that **e**_*a*_(*n*), **e**_*p*_(*n*), and $\hat{h}(n)$ are independent of **x**(*n*) in the steady state, we get

(74) E[epT(n)[U(n)UT(n)]−1ep(n)] =E[eaT(n)[U(n)UT(n)]−1ea(n)]  −γSL024E{TT(n)U+(n)U(n)T(n)}  +γSL024E[TT(n)T(n)].

Substituting (71) into the left-hand side (LHS) of (74), we have

(75) LHS=(1−μSL0)2E{eT(n)[U(n)UT(n)]−1e(n)}−(1−μSL0)E{eT(n)[U(n)UT(n)]−1v(n)}−(1−μSL0)E{vT(n)[U(n)UT(n)]−1e(n)}+E{vT(n)[U(n)UT(n)]−1v(n)}.

Moreover, substituting (70) into the right-hand side (RHS) of (74), we have

(76) RHS=E{eT(n)[U(n)UT(n)]−1e(n)}−E{eT(n)[U(n)UT(n)]−1v(n)}−E{vT(n)[U(n)UT(n)]−1e(n)}+E{vT(n)[U(n)UT(n)]−1v(n)}−γSL024E{TT(n)U+(n)U(n)T(n)}+γSL024E[TT(n)T(n)].

By combining (75) and (76), we get

(77) (2μSL0−μSL02)E{eT(n)[U(n)UT(n)]−1e(n)} =μSL0E{eT(n)[U(n)UT(n)]−1v(n)}  +μSL0E{vT(n)[U(n)UT(n)]−1e(n)}  +γSL024E{TT(n)U+(n)U(n)T(n)}  −γSL024E[TT(n)T(n)].

We also assume that the additive Gaussian noise **v**(*n*) is statistically independent of the input signal **x**(*n*). Thus (77) can be simplified as

(78) E{eT(n)[U(n)UT(n)]−1e(n)} =12−μSL0E{vT(n)[U(n)UT(n)]−1v(n)}  +γSL024(2μSL0−μSL02)E{TT(n)U+(n)U(n)T(n)}  −γSL024(2μSL0−μSL02)E[TT(n)T(n)].

Here, we also assume that the **U**(*n*) is statistically independent of **e**(*n*) at the steady state. Moreover, we use the definition of E[**e** ^*T*^(*n*)**e**(*n*)] = **E**|*e*_*t*_(*n*)|^2^ **S** [36], where

(79) $$\begin{array}{c} S\approx \{\begin{array}{cc} I, & \mu_{\text{SL}0}\;\;\text{is}\;\;\text{small}\\ 1\cdot 1^{T}, & \mu_{\text{SL}0}\;\;\text{is}\;\;\text{large}, \end{array} \end{array}$$

where $1^{T}=\left[\begin{array}{cccc} 1 & 0 & \cdots & 0 \end{array}\right]$ and *e*_*t*_(*n*) is the top entry of **e**(*n*) [36]. Then, the LHS of (78) can be rewritten as

(80) E{eT(n)[U(n)UT(n)]−1e(n)} ≈Tr⁡{E[eT(n)e(n)[U(n)UT(n)]−1]} ≈E|et(n)|2Tr⁡{S·E[[U(n)UT(n)]−1]}.

Similar to the calculation of (80), the first term in the RHS of (78) can be written as

(81) E{vT(n)[U(n)UT(n)]−1v(n)} =Tr⁡{E[vT(n)v(n)[U(n)UT(n)]−1]} =Qδv2Tr⁡{E[[U(n)UT(n)]−1]}.

In addition, the second term of RHS of (78) can be rewritten as

(82) γSL024(2μSL0−μSL02)E{TT(n)U+(n)U(n)T(n)} ≈γSL024(2μSL0−μSL02)δsgn⁡T(h)(hT+δ)2E{U+(n)U(n)}δsgn⁡(h)(h+δ)2.

Then the last term of the right-hand side of (78) can be expressed as

(83) γSL024(2μSL0−μSL02)E[T(n)TT(n)] =γSL024(2μSL0−μSL02)δsgn⁡T(h)(hT+δ)2δsgn⁡(h)(h+δ)2.

When the *μ*_SL0_ is small, we can get

(84) Tr⁡{S·E[[U(n)UT(n)]−1]} =Tr⁡{I·E[[U(n)UT(n)]−1]} =QNδx2.

Therefore, the MSE of the proposed SL0-APA with small step-size *μ*_SL0_ can be written as

(85) MSEsmall=12−μSL0δv2+γSL024(2μSL0−μSL02)δsgn⁡T(h)(hT+δ)2Nδx2QQRNδx2δsgn⁡(h)(h+δ)2−γSL024(2μSL0−μSL02)δsgn⁡T(h)(hT+δ)2Nδx2Qδsgn⁡(h)(h+δ)2=12−μSL0δv2+γSL024(2μSL0−μSL02)δsgn⁡T(h)(hT+δ)2×(R−Nδx2QI)δsgn⁡(h)(h+δ)2.

When the step-size *μ*_SL0_ is large, **S** ≈ 1 · 1^*T*^ [36]. In this case,

(86) $$\begin{array}{c} \mathrm{Tr}\left\{S\cdot \text{E}\left[{\left[U\left(n\right)U^{T}\left(n\right)\right]}^{-1}\right]\right\}=\frac{1}{N\delta_{x}^{2}}. \end{array}$$

Thus, the MSE of the proposed SL0-APA with large step-size *μ*_SL0_ can be written as

(87) MSElarge=12−μSL0δv2Q+γSL024(2μSL0−μSL02)δsgn⁡T(h)(hT+δ)2Nδx2QRNδx2δsgn⁡(h)(h+δ)2−γSL024(2μSL0−μSL02)δsgn⁡T(h)(hT(n)+δ)2Nδx2δsgn⁡(h)(h+δ)2=12−μSL0δv2Q+γSL024(2μSL0−μSL02)δsgn⁡T(h)(hT+δ)2×(QR−Nδx2I)δsgn⁡(h)(h+δ)2.

## 4. Results and Discussions

In this section, we present the computer simulation results to illustrate the performance of the proposed SL0-APA over a sparse multipath communication channel. Moreover, the simulation results for predicting the mean-square error of the proposed SL0-APA are also provided to verify the effectiveness of the theoretical expressions obtained in Section 3.2. In addition, the computational complexity of the SL0-APA is presented and compared with past sparsity-aware algorithms, namely, the ZA-APA, RZA-APA, and standard APA, NLMS algorithms.

### 4.1. Performance of the Proposed SL0-APA

**Figure 2:**
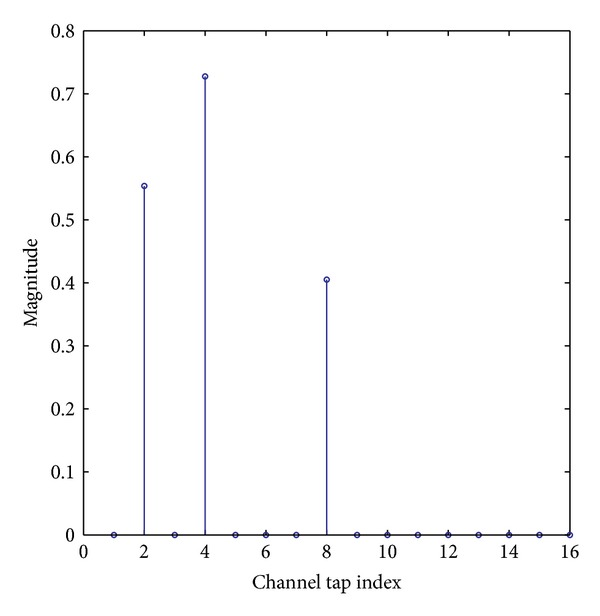
A typical sparse multipath channel.

Firstly, we set up a simulation example to discuss the convergence speed of the proposed SL0-APA in comparison with the previously proposed sparse channel estimation algorithms including the APA, ZA-APA, RZA-APA, and NLMS algorithms. In the setup of this experiment, we consider a sparse multipath communication channel **h** whose length *N* is equal to 16 and whose number of dominant taps *K* is set to two different sparsity levels, namely, *K* = 1, *K* = 4, similarly to [6, 22, 25, 26]. The dominant channel taps are obtained from a Gaussian distribution subjected to ||**h**||_2_^2^ = 1, and the positions of the dominant channel taps are random within the length of the channel. The input signal **x**(*n*) of the channel is a Gaussian random signal, while the output of the channel is corrupted by an independent white Gaussian noise **v**(*n*). An example of a typical sparse multipath channel with a channel length of *N* = 16 and a sparsity level of *K* = 3 is shown in Figure 2. In the simulations, the power of the received signal is *E*_*b*_ = 1, while the noise power is given by *δ*_*v*_^2^. In all the experiments, the difference between the actual and estimated channels based on the sparsity-aware algorithms and the sparse channel mentioned above is evaluated by the MSE defined as follows:

(88) $$\begin{array}{c} \text{MSE}\left(n\right)=10\;\log_{10}\text{E}\left\{{||h-\hat{h}\left(n\right)||}_{2}^{2}\right\}\left(\text{dB}\right). \end{array}$$

**Figure 3:**
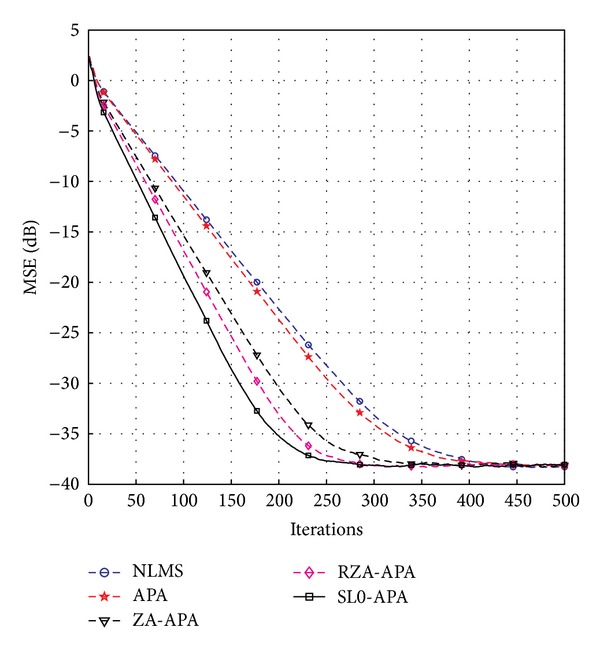
Convergence of the proposed SL0-APA.

**Figure 4:**
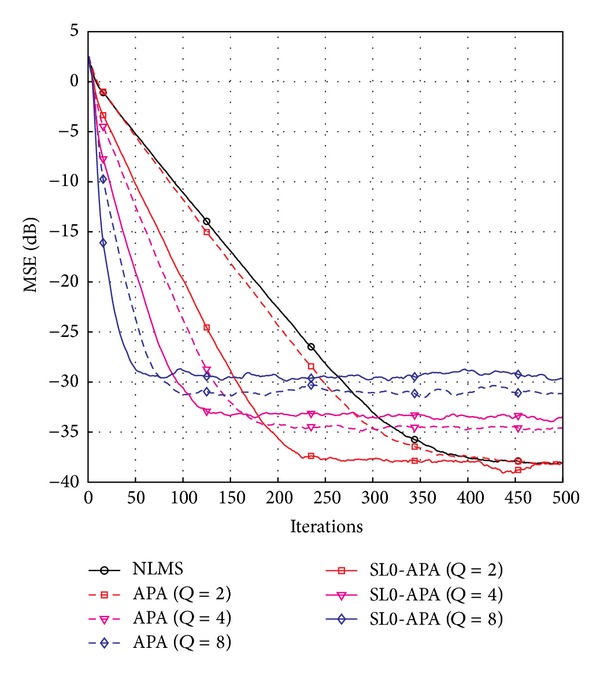
Affine projection order effects on the SL0-APA.

In this subsection, we aim to investigate the convergence speed and the steady-state performance of the SL0-APA. The simulation parameters used to compare the convergence speed while maintaining the same MSE are listed as follows: *μ*_NLMS_ = 0.25, *μ*_APA_ = 0.125, *μ*_ZA_ = 0.165, *μ*_RZA_ = 0.18, *μ*_SL0_ = 0.21, *γ*_ZA_ = 5 × 10^−5^, *γ*_RZA_ = 8 × 10^−5^, *γ*_SL0_ = 3 × 10^−6^, *ε*_RZA_ = 10, *δ*_SL0_ = 0.001, *Q* = 2, and *δ*_*v*_^2^ = 10^−3^, where *μ*_NLMS_ is the step-size parameter for NLMS algorithm. It can be seen from Figure 3 that our proposed SL0-APA possesses the fastest convergence speed compared to the previously proposed channel estimation algorithms at the same steady-state error floor. In addition, all the affine projection algorithms, namely, APA, ZA-APA, RZA-APA, and SL0-APA, converge much more quickly in comparison with NLMS algorithm, because the affine projection algorithms reuse the old data signal that is implemented by the use of parameter *Q*. Thus, we discuss the effects of the affine projection order *Q* for SL0-APA and compare it with the APA and NLMS algorithms. The computer simulation results with different values of *Q* are shown in Figure 4. It reveals that the convergence speed is improved by the increment of the affine projection order *Q*. However, the steady-state performance has deteriorated from *Q* = 2 to *Q* = 8. Thus, in our proposed SL0-APA, the affine projection *Q*, the step-size *μ*_SL0_, the regularization parameter *γ*_SL0_, and *δ*_SL0_ should be take into account to balance the convergence speed and the steady-state behavior.

**Figure 5:**
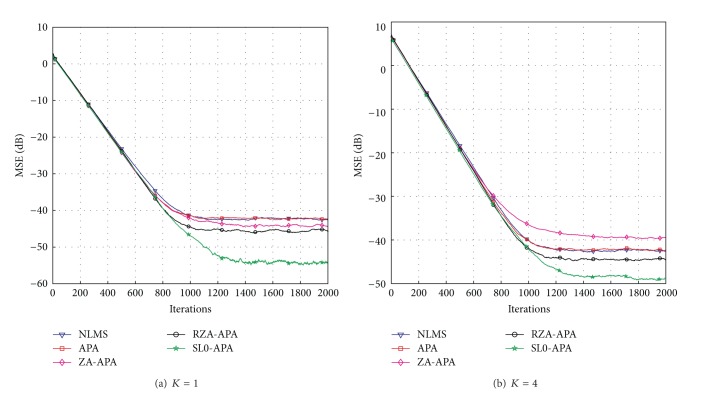
Performances of the SL0-APA with different sparsity levels for *Q* = 2.

**Figure 6:**
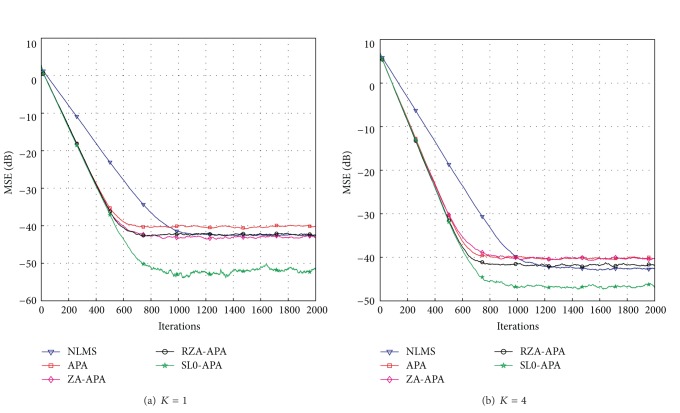
Performances of the SL0-APA with different sparsity levels for *Q* = 3.

Next, we show the effects of the sparsity levels on the steady-state performance of the proposed SL0-APA at *K* = 1 and *K* = 4. To obtain the same convergence speed, the simulation parameters used in this experiment are listed as follows: *μ*_NLMS_ = 0.095, *μ*_APA_ = *μ*_ZA_ = *μ*_RZA_ = *μ*_SL0_ = 0.05, *γ*_ZA_ = 5 × 10^−5^, *γ*_RZA_ = 8 × 10^−5^, *γ*_SL0_ = 4 × 10^−6^, *ε*_RZA_ = 10, *δ* = 0.01, and *δ*_*v*_^2^ = 10^−3^. We can see from Figure 5 that our proposed SL0-APA has the best steady-state performance compared to the ZA-APA, RZA-APA, APA, and NLMS algorithms. The SL0-APA can achieve 10 dB smaller MSE than the RZA-APA for *K* = 1 and *Q* = 2 shown in Figure 5(a). When the sparsity level *K* increases to 4, it is seen in Figure 5(b) that our proposed SL0-APA still outperforms other algorithms, while its steady-state error increases in comparison with that of *K* = 1. When the affine projection order increases to *Q* = 3, we can see from Figure 6 that the convergence speed is significantly improved compared to that of *Q* = 2 shown in Figure 5. However, the steady-state error is also slightly increased when the *Q* increases. Furthermore, our proposed SL0-APA still has the best convergence speed and lowest steady-state error.

**Figure 7:**
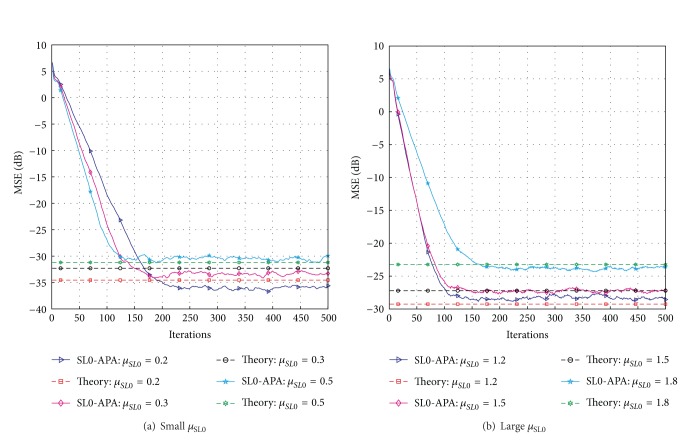
Steady-state MSE performance of the SL0-APA with different step-size *μ*_SL0_ for *K* = 4.

Finally, we use the theoretical expressions obtained in Section 3.2 to predict the mean-square-error (MSE) of the proposed SL0-APA with different *μ*_SL0_ and compare the theoretical results with the simulation ones. The MSE comparisons of the SL0-APA as a function of the step-size *μ*_SL0_ for the designated sparse multipath communication channel with the simulation parameters of *γ*_SL0_ = 4 × 10^−6^, *δ* = 0.01, *δ*_*v*_^2^ = 10^−3^, *Q* = 3, and *K* = 1 are shown in Figure 7. The theoretical results are obtained from (85) to (87) for small values of *μ*_SL0_ and large values of *μ*_SL0_, respectively, while the simulation results are obtained by averaging 50 independent trials. We can see that the simulation results exhibit good agreement with the theoretical expressions with different step-size *μ*_SL0_. In addition, we can see that the steady-state misadjustment between the computer simulation and the theory predicting is becoming larger with the decrement of the *μ*_SL0_ for small *μ*_SL0_ shown in Figure 7(a), but the steady-state error is becoming lower. For the large *μ*_SL0_, both the steady-state error and the convergence speed are deteriorated by the increment of the step-size *μ*_SL0_. Generally speaking, as *μ*_SL0_ increases, the MSE increases. Although a large zero attractor can help the SL0-APA to converge faster, it will lead to a higher misadjustment. Thus, in the most cases, we should choose the step-size *μ*_SL0_ carefully in order to balance convergence speed and steady-state performance.

### 4.2. Computational Complexity

In this subsection, we present the computational complexity of the proposed SL0-APA and compare it with the conventional sparsity-aware channel estimation algorithms, including the APA, ZA-APA, and RZA-APA. It is worth noting that when the affine projection order *Q* is equal to 1, these three affine projection algorithms converge to familiar NLMS, ZA-NLMS, and RZA-NLMS algorithms, respectively. Here, the computational complexity is the arithmetic complexity, which includes additions, multiplications, and divisions. We assume *K* nonzero taps in a sparse channel model as an FIR filter with *N* coefficients, and the order of these affine projection algorithms is *Q*. The computational complexity of the proposed SL0-APA and the relevant sparsity-aware algorithms are shown in Table 1.

From Table 1, we see that our proposed SL0-APA with the best steady-state performance and fastest convergence speed needs more calculations than the RZA-APA. The additional computational complexity comes from the continuous function for SL0 approximation, which can be reduced by proper selection of this continuous function. Furthermore, the complexity of all the APAs is higher than the NLMS algorithms. In addition, the sparsity property of the channel can also help to reduce the computational complexity of the proposed SL0-APA.

## 5. Conclusion

In this paper, we proposed an SL0-APA to exploit the sparsity of sparse channel and to improve the performance on both the convergence speed and steady-state error of the APA, ZA-APA, and RZA-APA. This algorithm is mainly developed by introducing a smooth approximation *l*_0_-norm, which has a significant impact on the sparsity due to the incorporation of SL0 into the cost function of the standard APA as an additional constraint. The improvement can evidently accelerate the convergence speed by exerting such additional regularization term on the zero taps of the sparse channel. Then, we provided a mathematical analysis for predicting the mean square error of our proposed SL0-APA. We also showed the convergence behavior and the steady-state performance in comparison with the standard APA and relevant sparsity-aware channel estimation algorithms. In summary, the simulation results demonstrated that the proposed SL0-APA with moderate computational complexity accelerates convergence speed and improves steady-state performance in a designated sparse channel.

## Figures and Tables

**Table 1:** Computational complexity.

Algorithms	Additions	Multiplications	Divisions
NLMS	3*N*	3*N* + 1	1
ZA-NLMS	*N* + 3*K*	*N* + 3*K* + 1	1
RZA-NLMS	*N* + 4*K*	*N* + 4*K* + 1	*N* + 1
APA	*NQ* ^2^ + *NQ* + *K* − *N* + O(*Q* ^3^)	*NQ* ^2^ + *Q* ^2^ + *NQ* + *Q* + O(*Q* ^3^)	*Q*
ZA-APA	*NQ* ^2^ + *Q* ^2^ + 3*NQ* − 2*Q* − 2*N* + 3*K* + O(*Q* ^3^)	*NQ* ^2^ + 2*Q* ^2^ + 3*NQ* + 2*Q* + *K* + O(*Q* ^3^)	*Q*
RZA-APA	*NQ* ^2^ + *Q* ^2^ + 3*NQ* − 2*Q* − 2*N* + 4*K* + O(*Q* ^3^)	*NQ* ^2^ + 2*Q* ^2^ + 3*NQ* + *Q* + *N* + 2*K* + O(*Q* ^3^)	*N* + *Q*
SL0-APA	*NQ* ^2^ + *Q* ^2^ + 3*NQ* − 2*Q* − 2*N* + 4*K* + O(*Q* ^3^)	*NQ* ^2^ + 2*Q* ^2^ + 3*NQ* + *Q* + *N* ^2^ − *N* + 2*K* + O(*Q* ^3^)	*N* + *Q*
